# Collection of Memoirs on Military Medicine, Surgery, and Pharmacy. Published Officially

**Published:** 1847-01

**Authors:** 


### Art. IV.

*Recueil de Mémoires de Médecine, de Chirurgie, et de Pharmacie Militaires; redigé sous la Surveillance du Conseil de Santé des Armeés. Publié par Ordre de S. Ex. le Ministre Secrétaire de l'Etat au Département de la Guerre.—Paris,* 1815-1846.

*Collection of Memoirs on Military Medicine, Surgery and Pharmacy. Published officially.—Paris,* 1815-1846. Fifty-eight volumes, 8vo.

In taking a review of the periodical literature of the present day, a class of publications which exists in France cannot fail to attract our attention, as being of a description to which we possess nothing analogous in this country. It consists of journals published under the control and at the expense of government, and consequently possessing in a great measure the character of official documents. Their importance as affording the means of disseminating information among particular classes of individuals, and of thereby leading opinion in some measure, by an appeal to reason, is deserving of deep consideration. This would involve a greater amount of space and leisure than we can spare, and would moreover lead us into a discussion of too general a character to be suited to the pages of a medical journal. Among the periodicals of the kind referred to, however, is one which is recognized by the French government as the official organ of the army medical department, and which was instituted with a view to promote sound instruction among its officers. We propose in the following pages to give a brief sketch of the origin of this journal, and to notice one or two of the most striking papers which have appeared in its pages.

I. In 1763 Dr. Richard de Hautesierck, inspector of military hospitals, pointed out to the Duc de Choiseul, then minister of war, the advantages which would accrue to the medical department of the army, from calling upon the surgeons attached to hospitals to give a regular account of their practice, and to correspond on the subject with the inspector-general, who should be empowered to publish the results of that correspondence. The minister authorised Dr. Richard to carry out his plan, and to collect and publish at the expense of government any interesting observations and rare cases which might thus be communicated to him. In 1766 he accordingly brought out a quarto volume, entitled, 'Recueil d'Observations de Médecine des Hôpitaux Militaires,' wherein, after laying down the plan on which the journal was to be in future conducted, he pointed out the necessity of studying the medical and physical topography of the countries commonly occupied by the troops, and especially the salubrity or insalubrity of the various garrison towns, barracks, prisons, and hospitals. He also gave several reports of cases, descriptions of epidemics, some topographical memoirs, particularly of the towns of Montpellier, Châlons-sur-Saône, Toulon, Lille, Bitche, and Strasburg, and a formulary of prescriptions for the use of the military hospitals. The gratuitous distribution of this work excited the zeal of the medical officers of the army, and increased the amount of correspondence on these subjects. In 1772 a second volume was published, which contained four memoirs on topography, five on epidemic diseases observed in France between 1764 and 1770, with many medical and surgical cases. Dr. Richard, for his services, received the riband of St. Michel, and was created Baron de Hautesierck.

In 1781 an ordonnance was published on the subject of the medical department of the army, by which, among other things, the 'Journal de Médecine, de Chirurgie, et de Pharmacie Militaires' was established; it was to appear every three months, and to be compiled by a retired consulting physician of the army. The object of this journal was to promulgate facts and opinions relative to the preservation of the health of soldiers, or to the successful treatment of their diseases, and nothing foreign to the medical department of the army, or of the military hospitals, was to be inserted. The first volume was published in 1782, and it continued to appear regularly till 1789, forming seven octavo volumes.

The changes in the administration of the army by the council established by the minister of war in 1788, caused the publication of the journal to be suspended. It was not intended to suppress it altogether, but the new directory of the hospitals announced, in 1789, that it would no longer be brought out at stated times as a periodical work. From this date till 1801, the instability of affairs in France, and the numerous calls of duty on the council of health of the army, prevented the preparation of another volume. In that year several officers were appointed to prepare a summary of the most important papers which had been collecting during the preceding twelve years, but before this was completed their services were required with the grand army. Nothing further appears to have been done till 1815, when the journal was re-established, MM. Biron and Fournier Pescay being appointed the editors. It was at first brought out in bimonthly numbers; but this having been attended with many disadvantages, the editors resolved in 1817 to publish it for the future in half-yearly volumes, and the title was at the same time changed to that which it at present bears.

The minister of war, in his letter to the inspectors of hospitals in 1815, states the object of the journal to be, "to diffuse sound instruction among the medical officers of every rank, and to communicate to them without delay the discoveries which shall be made in the theory and practice of the healing art. All the medical officers are called upon to contribute materials to the journal. The publication of their labours will have the double advantage of being useful to the service, and of maintaining among all a noble emulation. In short, this journal will become a depôt where each one may treasure up the result of his researches and the discoveries he may have made."

To obtain the materials necessary for carrying on this work, the principal medical officers of hospitals and the surgeon-majors of regiments were directed to forward monthly reports embracing all subjects relating to the health of the troops, either in the prevention or treatment of disease; they were also to give a detailed history of rare cases of disease among the soldiers; an account of any epidemics, with their probable causes and most successful treatment; meteorological observations, &c. The principal medical officers of hospitals were likewise to transmit quarterly, numerical returns of the admissions and deaths, and of the diseases by which these were caused. If these were furnished regularly, but little use appears to have been made of them, which we the more regret, as army medical officers possess opportunities of compiling satisfactory reports which rarely fall to the lot of the medical profession in civil life.

The editors being fully impressed with the importance of the study of military hygiene, called the attention of the medical officers to the advantages to be derived from a careful examination of the "rules and precepts relating to the preservation of the health of soldiers, and to the most suitable means for removing or diminishing the fatal influence of the numerous causes of disease to which they are exposed both in peace and war." M. Biron, in the second volume of the journal, published a valuable memoir on this subject, in which he directed attention to the principal objects of study. These he arranged under seven general heads:—1. Of the choice of the soldier—his physical and moral qualities—the influence of military discipline on the recruit. 2. Of the diet of soldiers. 3. Of the clothing of the troops. 4. Of their quarters:—*a*, barracks; *b*, military prisons; *c*, hospitals; *d*, camps and bivouacs. 5. Of marches, exercises, and military works; the influence of, *a*, victories; *b*, retreats; *c*, captivity. 6. Duties of officers—discipline and habits of soldiers—inculcating the maxim "qu'il faut le défendre contre lui-même, et lui faire du bien malgré lui." 7. Of the duties of surgeon-majors of regiments.

Fifty-eight volumes of this journal have now been published, a monument of the industry of the medical officers of the French army, and of the zeal and good sense of the council of health. The subjects chiefly treated, besides numerous interesting cases in medicine and surgery, are hygiene, medical topography, histories of epidemics among the troops, clinical reports from various military hospitals, surgical histories of campaigns, reviews of works on military medicine and surgery, biographical notices of deceased medical officers of the army, extracts from the addresses to the pupils of the military hospitals at the annual *concours*, and the names of the successful candidates at these *concours*.

It is much to be regretted that a work of a similar nature has never been established in the British service. The Army and Navy Statistical Reports already published, furnish much interesting and valuable information regarding the topography of the military stations, and the prevalence of disease and mortality among the soldiers and sailors employed in several of our colonies and dependencies ; but the observations on the other subjects above mentioned pass unrecorded, or, if recorded, are at least very rarely made available to the use of the profession. That there is no lack of zeal in the collection of materials, let the accumulated documents at the Army and Navy Boards bear witness. But for what purpose such accumulation, if the papers are to lie untouched, their contents uncommunicated to those likely to benefit by them? These reports and observations, contributed during upwards of a quarter of a century, by officers employed in all parts of the world, many of whom have been men of considerable literary and scientific acquirements, doubtless contain much that would interest the statesman, the ethnologist, and the natural philosopher, that would conduce to the advancement of medical science, and that would prove useful and instructive to the junior officers of the department. It is a source of much regret that no synopsis of the best papers thus immured in the record rooms of the Boards has ever been published, or that the materials of so large a collection have not been methodically digested, arranged, and made easily accessible to the profession. We understood, some years ago, that it was in contemplation to condense and publish the more important documents at the Army Medical Board ; and although this plan seems for the present in abeyance, we trust it has not been finally abandoned.

It has been remarked of this country that we possess no military literature ; we are, certainly, by no means rich in military medical literature. The field of observation and exertion is wide and inviting ; and the opportunities enjoyed by officers of acquiring information, in regard to climate, and the diseases of troops in the stations where they are employed, are in many respects excellent. The medical department of the army, however, have much cause to lament that so few of their body raise a memorial of their talents, zeal, and industry, by publishing the result of their extended and varied experience. From the communications of those who have enjoyed superior opportunities of observation and of acquiring knowledge, we may naturally expect to gain much useful information upon the influence of climate, and the means of preserving the health of soldiers; and we think it will be generally admitted that those officers who have attained high rank, and filled desirable appointments, owe it as a duty to their country, and to the service in which they have gained their honours, to place upon record, and thus render available, the results of their long experience. Sir John Pringle and Dr. Jackson, by their highly valuable works, established a claim to the perpetual gratitude of the army, and left behind them imperishable monuments of their great talents and indefatigable industry, and an example worthy of being followed by their successors.

But in thus asserting the claims of the army and navy upon individual officers, we by no means wish to underrate their claims upon Government.

M. Begin, in an address delivered at the annual *concours* of the Military Hospital of the Val de Grâce, remarks—

> "If the surgeon in civil life commits any mistakes, they are isolated, injure only the individuals, and are reproduced but at long intervals; while the errors of the military medical officer, acting upon masses, may assume the nature of a public calamity. The first, when his practice is irrational, or very unfortunate, loses the confidence of his patients, and the evil checks itself; the other, armed with military rank, pursues his course steadily, while the men who are intrusted to his care cannot escape from his treatment. An extreme caution, therefore, is imposed upon the army medical officer. While adopting the progressive improvements of science, he ought never to forget that our soldiers cannot, guiltlessly, be made the subjects of rash experiments."

Such being the case, it surely becomes the duty of Government to afford to medical officers the means of acquiring early and authentic information on all subjects connected with the preservation of the health, or the treatment of the diseases of soldiers; since, from being scattered over the face of the globe, they have not the same opportunities as their brethren in civil life of becoming acquainted with the recent discoveries in medicine.

The 'Journal de Médecine Militaire' does not appear to us to have accomplished all the objects for which it was instituted, or to have realized the anticipations which might reasonably be formed of its usefulness. But this has resulted rather from the manner in which it has been conducted than from any fault in its original constitution. Indeed, we are inclined to think it is mainly owing to the original plan having been neglected, and a faulty system substituted. For instance, the reviews of new works on medicine and surgery have been almost altogether omitted, and the biographical sketches are so few as to justify the inference that this branch is considered of very secondary importance. The compilation of numerical returns from the hospitals, one of the most striking and important features in the original plan, has never been carried out, while an undue proportion of the volumes has been devoted to the history of cases. Besides, the distribution of the journal is confined, we understand, to the principal medical officer of each hospital and to the surgeon-majors of regiments. This appears rather to savour of sending coals to Newcastle; for if the object of the journal be the diffusion of sound instruction among the medical officers of the army, it would seem most natural to place it in the hands of the juniors, who have yet much to learn of the practical part of the profession, and not confine the distribution to the seniors, whose practice may naturally be supposed to have been matured by long experience, and many of whom would not alter their own views were all the books written since the days of Solomon arrayed against them. Whatever may be the reason, it is certain that the journal is not in high favour among the medical officers, whose best papers generally appear in the ordinary medical periodicals, and not in this, their official organ.

II. We shall now proceed to extract a few of the most striking observations which we have met with in looking over this work. The first we shall submit is a contribution to the statistics of gunshot wounds, from a ' Surgical History of the occurrences which took place at Lyons, during and after the six days of April" (1834), by Dr. Laroche, P. M. O. of the Military Hospital there (vol. xxxvii).

| GENERAL TABLE OF WOUNDS. | | | | Admitted. | Died. | Recovered. |
|---|---|---|---|---|---|---|
| Gunshot Wounds | of the Head | Cranium | | 12 | 4 | 8 |
| | | Face | | 14 | 2 | 12 |
| | of the Trunk | Neck | | 8 | 2 | 6 |
| | | Shoulders | | 8 | 3 | 5 |
| | | Chest | penetrating | 6 | 5 | 1 |
| | | | non-penetrating | 12 | 2 | 10 |
| | | Abdomen | penetrating | 5 | 5 | 0 |
| | | | non-penetrating | 9 | 0 | 9 |
| | | Pelvis | penetrating | 7 | 5 | 2 |
| | | | non-penetrating | 13 | 0 | 13 |
| | of the Upper Extremities | | Arms | 20 | 8 | 12 |
| | | | Forearms | 5 | 1 | 4 |
| | | | Hands | 10 | 1 | 9 |
| | of the Lower Extremities | | Thighs | 39 | 9 | 30 |
| | | | Legs | 41 | 8 | 33 |
| | | | Feet | 22 | 2 | 20 |
| | of the Articulations | | Elbow-joint | 4 | 3 | 1 |
| | | | Hip-joint | 1 | 1 | 0 |
| | | | Knee | 7 | 5 | 2 |
| | | | Foot | 3 | 3 | 0 |
| Fractures | of the Arm | | | 1 | 0 | 1 |
| | of the Leg | | | 1 | 0 | 1 |
| Slight contusions | | | | 29 | 0 | 29 |
| | | | | 277 | 69 | 208 |

This table does not show a very favorable result of the treatment, 1 in 4 of all the cases admitted having died. It would be unfair to compare this with the results of wounds received in action, because, in street fighting, from the greater proximity of the combatants, the nature of the weapons employed, and the advantage the populace have in firing from windows, &c., the injuries received by the soldiers are usually of a very severe description—the only correct standard of comparison would be that of wounds inflicted under similar circumstances. In the 30th volume of the journal, M. Hippolyte Larrey has given a surgical history of the cases admitted into the Hospital of the (ex) Guard in Paris, during the three days of July, from which it appears that 20 died out of 266 admitted. He has not, however, given any tabular statement of the nature of the wounds, and we are consequently unable to ascertain in what particulars they differed from those reported by M. Laroche. This is a point of great importance in making a comparison, as will be seen on examining the results of the wounds of the chest, abdomen, and pelvis—15 having died out of 18 cases of penetrating wounds, while only 2 died of 34 in which the cavities were not entered. Wounds of the articulations also proved remarkably fatal, 12 having died out of 15 admitted.

The following were the results of the capital operations performed in the hospital at Lyons:

| | | | Died. |
|---|---|---|---|
| Trepanning | | 1 | 1 |
| Amputations | at Shoulder-joint | 2 | 1 |
| | of Arm | 7 | 6 |
| | of Forearm | 1 | 0 |
| | of Thigh | 4 | 3 |
| | of Leg | 5 | 3 |
| Ligature of Femoral Artery | | 3 | 1 |

M. Larrey has given the details of 16 amputations which were performed in the Hospital of the Guards, from which we have obtained the following results:

| | | | Died. |
|---|---|---|---|
| Amputations | at Shoulder-joint | 2 | 1 |
| | of Arm | 4 | 2 |
| | of Thigh | 5 | 1 |
| | of Leg | 5 | 3 |

Of these, 8 were primary, and 2 died; while of 8 secondary amputations, 5 terminated fatally.

The mortality arising from disease in an army on active service is usually *much greater* than that caused by the immediate casualties of the battle-field, and the success of military operations often depends on the General being able to bring up his forces unencumbered with sick, and in a condition to go through the arduous fatigues of a campaign. It is, therefore, one of the most important, as it is one of the most difficult duties of army surgeons, to prevent disease and maintain the troops in a state of health and efficiency. This can only be done by a careful examination into the causes of sickness among them, by diligent attention to the subject of their diet, dress, quarters, drills, &c., and by prompt treatment of disease the moment it manifests itself. The following heads of recommendations by the medical officers, for the preservation of the health of the troops, promulgated at Burgos in May, 1823, when the French army was advancing into Spain, are taken from a history of that campaign, by M. U. Coste, vol. xvi:

"1. The soldiers always to wear their *cloth* trousers.

2. Not to be permitted to undress, as they usually do, on arriving at the halting-place or at their bivouac in the evening.

3. To wear their great-coats whenever the air is chilly, and when they are not on the march or working.

4. In wet weather, to halt for the night on ground a little elevated, sloping, and sheltered from the wind.

5. To increase in such cases the number of fires, and to keep them up till the time of starting.

6. In wet weather, to make an additional distribution of brandy on starting, and on arriving at the bivouac.

7. In hot weather, to march the troops early in the morning, or in the evening, resting during the middle of the day in valleys or sheltered places.

8. If linen trousers are permitted to be worn, to make the soldier put on a girdle of cloth or woollen stuff round the belly,

9. To make frequent halts, selecting places where the water is good, and to take care that the men do not drink cold water when greatly overheated.

10. To give them regular exercise when in cantonments.

11. To be careful that their clothes and shoes are kept up and repaired.

12. When compelled to use bad water, to mix a little vinegar or brandy with it.

13. In marshy places, to encamp, if possible, on the high ground."

A most important branch of the study of military hygiene is medical topography, as by it we are led to consider the influence of the various physical agents upon the health of the soldier, and to remove him when practicable from exposure to the causes of endemic diseases. This subject has received a considerable share of attention from the medical officers of the French army, and in this journal we find many excellent topographical memoirs. As these have been mostly drawn up on a uniform plan, it may not be amiss here to quote from a paper, by M. Estienne, the subjects to which the attention of the officers was especially called in preparing them :

"1. To point out the nature of the soil of the country to be described.

2. The longitude, latitude, and general description.

3. The prevailing winds. Are these proper to certain seasons, to certain determined periods ? What are these periodic winds ?

4. What are the principal physical qualities of the water of the rivers, ponds, and wells ? What influence do they exert on vegetation, and on the health of man and animals ?

5. What trees, shrubs, and other plants, particularly culinary and medicinal, grow in the places described ?

6. What grains are cultivated ? How are they cultivated ? And what are their diseases ?

7. To examine carefully and describe the numerous medicinal substances exported from the country, particularly to France.

8. The animals of all classes peculiar to the country. To collect as much information as possible regarding the domestic animals which lighten and share the labour of man.

9. Finally, to make known the general temperament of the inhabitants, their food, drink, clothing, the construction of their houses, their habits and manners ; to point out the most ordinary diseases of the children, men, girls, and women, and the customary mode of treating them ; to state at what period menstruation begins and ends ; if fecundity is considerable ; and what is the ordinary term of life."

In the later numbers of the journal are many topographical memoirs of the various stations in Algeria, which cannot fail to prove useful to the medical officers sent to join the army there. The numerical information, however, which is afforded regarding their salubrity is very scanty and very defective. It is probable that this information may be withheld from motives of political expediency, but what is given is very imperfect, the details relative to the French soldiers being mixed with those of the native auxiliaries, the strength of the troops being rarely stated, or other important omissions existing, which render the observations of little value to medical science.

Those who have studied the effects of a tropical climate upon the European, compared with the African or Asiatic, as illustrated by the Statistical Reports on the Health of the Army, will at once comprehend how uncertain must be the results derived from returns in which the two are mixed, while no means are furnished whereby to separate them, nor any estimate given of the relative numbers of each class. This remark is particularly applicable to the French army in Africa, because the principal diseases giving rise to sickness and mortality there are of endemic origin, and consequently of that description in which the difference is most strikingly marked. The deaths in hospital of all the troops, in 1841, amounted to 104 per 1000 of the strength. The only return we have met with relating to French soldiers alone, is one giving the amount of sickness, mortality, and invaliding in the 55th Regiment, at Bona, during the first year of its service in Africa. From this it appears that, in twelve months, they lost 250 per 1000 of the strength by death, and that a similar number were sent home to France as invalids, thus reducing the regiment, in that short period, to half its original numbers.

We had intended to have gone at some length into the subject of the sanitary condition of the army in Algeria, but, as all the returns are open to the very serious objections above noticed, and as all the deductions from them must consequently be viewed with mistrust, we have been reluctantly obliged to give up this interesting investigation.

In the fifty-fourth volume is a long and interesting paper by Dr. Casimir Broussais, on an epidemic of cerebro-spinal meningitis, which prevailed among the different garrisons in France from 1837 to 1842, and which appears to have been a disease of great intensity. We briefly noticed this epidemic in a previous number, but, as the present paper is compiled from the reports sent to the Council of Health by the military medical officers, and therefore comprehends a description of the progress of the disease through France, at least as it appeared among the troops (and it was chiefly confined to them), we shall condense the principal facts contained in it. It commenced at Bayonne, in 1837, among the military, and soon spread into Les Landes, many cases occurring among the inhabitants of the communes surrounding Dax. Thence it extended to Bordeaux, and in the same year to La Rochelle, in both of which places it was confined to the garrison. It then suddenly appeared at Versailles and St. Cloud, where it raged from 1839 to 1842. From Versailles it spread eastwards to Caen and Cherbourg, in 1840 and 1841; westwards to Metz, Strasbourg, Nancy, and Colmar, from 1839 to 1842; and southwards to Laval, Le Mans, Château-Gonthier, Tours, Blois, and Joigny, and finally appeared in the neighbourhood of Rambouillet. From La Rochelle it reached Poitiers in 1840, Lorient in 1841, and Ancenis and Nantes in 1841 and 1842. In all these places the disease was chiefly confined to the military.

But, while it thus extended in a northerly direction, it also spread among the garrisons to the west of Bayonne, appearing in 1837 at Narbonne and Foix, in 1838 at Toulon, and in 1839 at Nîmes. It prevailed at Avignon in the winter of 1839-40, and again in the following year; at Montbrison in 1840; and at Lyons in the winter of 1841-2. It appeared also at Perpignan in the winter of 1840-1, and the following year at Aigues-Mortes.

The progress of the epidemic was not marked by regularity, nor did it pursue a steady course from one garrison to another. Occasionally it appeared at a distant point, from which it sometimes returned to places it had passed over, while at other times it remained stationary for a considerable period. In some garrisons the disease did not prevail as an epidemic, but merely a few sporadic cases occurred; in others it appeared to become naturalized, and to take an endemic character.

The disease, as has been already stated, was confined chiefly to the military, but in a few instances extended its ravages also to the civil population; for instance, at Strasbourg, in 1841. Wherever it appeared in an epidemic form it was most fatal; upwards of half the cases reported having terminated in death. This is fully borne out by the following summary of the result in such of the garrisons as furnished numerical returns:

| | Cases. | Died. |
|---|---|---|
| Bayonne, January to March, 1841 | 28 | 21 |
| Versailles and St. Cloud, first epidemic, February to June, 1839 | 156 | 69 |
| ,, second epidemic, 1841, and first quarter of 1842 | 53 | 39 |
| Caen, 1840 | 10 | 4 |
| Cherbourg, second quarter of 1841 | 2 | 2 |
| Metz, November, 1839, to March, 1840 | 40 | 22 |
| Strasbourg, November, 1840, to May, 1841 | 184 | 108 |
| Nancy, January to August, 1841 | 28 | 8 |
| Colmar, February to April, 1842 | 7 | 5 |
| Laval, March, 1840, to March, 1841 | 69 | 44 |
| Le Mans, second epidemic, 1840 | 9 | 3 |
| Blois, January and February, 1841 | 12 | 4 |
| Poitiers, December, 1840, to February, 1841 | 20 | 8 |
| Ancenis, December, 1841 | 12 | 4 |
| Foix, April, 1837 | 16 | 6 |
| Montbrison, September to December, 1840 | 47 | 16 |
| Perpignan, October, 1840, to April, 1841 | 50 | 28 |
| Lyons, February to March, 1842 | 9 | 4 |
| Aigues-Mortes, November, 1841, to March, 1842 (civil population) | 160 | 120 |
| Strasbourg, January to December, 1841—cases reported among the civil population | 150 | 90 |

Thus it appears that in the returns furnished to the Council of Health of the Army by the medical officers, 1062 cases are recorded, of which 605 proved fatal. It is much to be regretted that the numerical details respecting this disease are not more comprehensive and more complete. M. Broussais makes some judicious remarks on the necessity of *systematic* investigation; many of the results forwarded to the council being unavailable, from the imperfect manner in which the facts are recorded. These remarks are well worthy of the consideration of the medical officers of the French army, as applicable not only to the present instance, but to almost all the statistical papers in the journal under review; and we believe that a perusal of them by the officers of our own army and navy would not be a work of supererogation. It is to incomplete observations we owe the existence of a host of *false* medical facts.

The disease is stated to have occurred chiefly among recruits and young soldiers. Several statistical documents are adduced in support of this; but they are of no value, as they merely show the numbers attacked in each year of service, without giving the strength out of which the cases occurred. From such data it is obviously impossible to estimate its relative prevalence among the different classes of troops. The predisposing causes of the disease are alleged to have been fatigue, crowded state of the barracks, those excesses into which young men are apt to run on first joining the army, with excessive drill, nostalgia, and the excitement attending the change from civil to a military life. It is said to have prevailed especially in the months of January, February, March, and April—periods corresponding with the arrival of the conscripts at their regiments. No meteorological phenomena were observed, which could account for the prevalence of the epidemic; nor was there any greater amount of cerebral disease during these years. No grounds exist for supposing that the disease was contagious.

The epidemic meningitis presented itself under two forms: 1st, of excitement, and, 2d, of collapse.

1. That of *excitement.* In this form there was a strong, quick pulse, sparkling eyes, red face, quickness of speech, headache or neckache, occasionally convulsive movements, and the patient made frequent complaints. Consciousness sometimes remained unimpaired, while at others there was temporary delirium, alternately disappearing and recurring. This at length terminated in the stage of collapse.

2. The form of *collapse* was characterized by general collapse, insensibility, coma. This, however, did not come on suddenly. The patients at first complained of fatigue, malaise, prostration, listlessness, and were often accused by their comrades of laziness and shamming. Dull, heavy headache followed; they lay with their eyes half closed; their speech was slow and painful; pulse slow and small; they had confusion of ideas, and vertigo. Coma was generally not complete; but although the patient could be roused, he answered questions with difficulty.

Although this form was usually the consequence of that of excitement, it was often developed as the primary stage of the disease. Both forms ran into a typhoid state, and sometimes became complicated with paraplegia or hemiplegia. Rachialgia was an invariable premonitory symptom. In the course of the disease marked intermissions occasionally were met with. Many cases, which were slight in appearance, terminated unexpectedly in death. Towards the end of the epidemic, the disease usually assumed a milder character than at the commencement.

On examination of the bodies after death, the membranes of the brain and spinal cord were found to present all the usual appearances of acute inflammatory action, from those of simple sanguineous injection and thickening up to serous or purulent infiltration. "The existence of a coating of false membrane in layers, flakes, or patches, has everywhere been the characteristic lesion."

The remedies employed have been general and topical bloodletting, ice to the head, sinapisms and vesicatories to the extremities, stimulating liniments, and the actual cautery to the spine. Emetics and purgatives, calomel and opium, and mercurial frictions, and, lastly, quinine.

Large bleedings in the early stage of the disease were the only means of treatment attended with success. The amount of blood to be drawn was measured by the effect upon the pulse, and not by the quantity. Unless treated thus energetically in the commencement, the patient had a very slight chance of recovery. In many of the cases the blood did not show the buffy coat. After the stage of excitement had passed, blisters to the head were found to be attended with benefit.

Such is a brief recapitulation of the circumstances narrated by M. Cas. Broussais relative to the progress, nature, and treatment of this remarkable epidemic—remarkable for the insidious manner of its attack, its extremely fatal character, unless treated most actively and at once, and its being confined almost exclusively to the military. We have seen one or two cases having a strong resemblance to those related in this memoir, but they occurred sporadically; and we are not aware of an epidemic of this nature having ever been observed in this country.

Perhaps we cannot conclude our present remarks more appropriately than by the following quotations from addresses delivered to the students at the Military Hospital of the Val de Grâce. These prove sufficiently that, although in the official journal of the medical department, the aid of statistical investigation has been greatly overlooked, many of the officers are fully aware of its importance; and we trust, ere long, to have it in our power to notice an increased attention to this branch of science.

"We ought undoubtedly," says M. Sedillot, "to value the truths which antiquity has handed down to us, and not to despise the experience of ages; but it is also necessary to apply to them the system of rigorous analysis which characterizes our era. . . . . . . We know that the mortality of the army weighs particularly upon the young conscripts; that it is increased by the change of garrisons; that it is greater in some places, as in Paris, or in localities affected by epidemics, than in others; but medicine has scarcely contributed to these results; they almost all belong to the labours of the administrative department. And if we are asked what are the most common diseases, and in what proportion do they attack soldiers, according to their age, length of service, distribution, their former profession, and a thousand other circumstances, we can only give approximative data, which might very easily be rendered certain, if we collected observations of the diseases. What one man could not do, several, by their co-operation, might easily accomplish. Our profession does not admit of repose; and in the intervals of practice we ought to strive unceasingly to increase our knowledge, and to extend the sphere of our usefulness."

"In military medicine, especially," says M. Begin, "statistics ought, as regards the modes of recruiting, the race of men, their regimen, drill, clothing, dwellings, removal from one climate to another, to lead to precise results, which it would be impossible to procure with the same degree of certainty in any other way. Figures, as has been often remarked, are inflexible, and cannot speak falsely; but we must exert ourselves to collect them, and group them according to their analogies; we must require from them only legitimate conclusions, and must bear in mind that the greater the numbers the more surely will the errors arising from negligence, or individual accidents, be cancelled, and truth predominate in the general result."

